# Age Estimation by Kvaal's Method Using Digital Panoramic Radiographs in the Saudi Population

**DOI:** 10.7759/cureus.23768

**Published:** 2022-04-02

**Authors:** Hosam S Alharbi, Ahmad M Alharbi, Abdulmajeed O Alenazi, Shaul Hameed Kolarkodi, Ramy Elmoazen

**Affiliations:** 1 Dentistry, Qassim University, Buraidah, SAU; 2 Dentistry, Almustaqbal University, Buraidah, SAU; 3 Maxillofacial Surgery and Diagnostic Science, Qassim University, Buraidah, SAU; 4 Dentistry, Eastern Finland University, Finland, FIN

**Keywords:** forensic dentistry, digital panoramic radiographs, saudi population, kvaal's method, age estimation

## Abstract

Introduction

In forensic literature, estimating an individual's age has garnered a lot of attention. With increasing age, the size of the dental pulp cavity shrinks as a result of secondary dentin deposits. This could be used as a measure of age. Aside from morphological approaches, radiological approaches might be used to analyze this regression shift. Kvaal's method calculates the chronological age of individuals based on the age-pulp size relationship on periapical dental radiographs.

Purpose

This study aims to use Kvaal's method to estimate the chronological age of patients using digital panoramic radiographs and verify the validity of regression equations proposed by Kvaal et al. in the Saudi population.

Material and methods

A total of 74 digital orthopantomograms were randomly selected from Qassim University Dental Clinic in Saudi Arabia, ranging in age from 18 to 64 years (mean age 32 years). The radiographs were taken between 2018 and 2021 according to inclusion and exclusion criteria.

Results

When the Kvaal technique was applied to Saudi members, there was no statistically significant discrepancy between the estimated and chronological ages. The coefficient of determination R2 was highest when three mandibular teeth were evaluated together (0.752).

Conclusion

The most accurate indicator for age assessment was "M" (mean worth, all things considered) and "W L" (contrast among "Width" and "Length").

## Introduction

In forensic science, age estimation, particularly age estimation utilizing teeth, plays a significant role in identifying unknown human bodies. The emergence of second molars is thought to have been used by the Romans to assess draught eligibility. Since the early nineteenth century, age assessment based on teeth has been professionally examined and implemented [1]. Thomsen utilized the rise of the first molar teeth to confirm the children's age in 1836 because, under English law, kids younger than seven couldn't be accused of a crime. Furthermore, in 1889, Lacassagne utilized grown-up teeth to decide the age of a dead individual [2]. Gustafson distributed an adult age estimation approach dependent on six age-related histological changes in teeth (optional dentin connection, whittling down, periodontitis, cementum juxtaposition, root resorption, and root straightforwardness) in 1950 [3]. Johanson isolated the Gustafson strategy's six classifications and proposed another condition for age assessment utilizing teeth in 1971, which has in this way been generally utilized for age assessment of the deceased [2,4].

Moreover, some age estimation strategies depend entirely on root clarity and periodontal downturn while others incorporate amino acid racemization of dentin, which is exceptionally exact [5]. While building up the age of living individuals, dental age assessment strategies that require tooth extraction are not useful. Therefore, noninvasive strategies have been grown, for example, degenerative changes in the pulp cavity on dental radiographs. Kvaal et al. distributed another age assessment approach dependent on periapical radiographs of alive Norwegian patients in 1995 [6]. The analysts analyzed the length and width of teeth and pulp cavities to consider the connections among ages and measurements. They created regression equations dependent on the study. The Kvaal age assessment approach has been utilized for all-encompassing radiographs in later examinations, albeit the outcomes have been blended. By refining the Kvaal age assessment approach, Paewinsky et al. introduced an age assessment condition utilizing the broadness of pulp cavities [7].

## Materials and methods

A total of 74 digital orthopantomograms were randomly selected from the University Dental Clinic in Saudi Arabia, ranging in age from 18 to 64 years (mean age of 32 years). The radiographs were taken between 2018 and 2021.

Patients with a maxillary central incisor, maxillary lateral incisor, maxillary second premolar, mandibular lateral incisor, mandibular canine, and mandibular first premolar should meet the inclusive measures. The teeth in the oral cavity that were without morphological abnormalities and had fully erupted clinical crowns were additionally in consideration standards.

Damaged teeth, malposed teeth, or teeth with radiopaque fillings, caries, and pathologic cycles in the apical bone were barred. Pregnant ladies, people with irregular hormonal characteristics, chemical substitution treatment, diabetes, or kidney disease were likewise remembered for exclusive standards.

All of the subjects who took part in the study signed an informed consent form. It was ensured that the data would only be utilized for scientific purposes.

Utilizing VistaScan DBSWIN programming (Dürr Dental, Bietigheim-Bissingen, Germany), estimations were taken on a normalized digital panoramic encompassing radiograph utilizing Kvaal's methodology for the six teeth that are under investigation. The reference focuses on the photos of the teeth that were characterized utilizing the mouse-driven pointer. The following estimations were taken: Length of the root that is present on the mesial side, starting from the cement enamel junction extending towards the root apex, and width of the root at different levels such as a, b, and c.

An individual observer completed the entirety of the estimations.

To estimate blunders brought about by contrasts in the amplification of the picture on the radiograph, similar tooth length and width ratios were resolved.

**Figure 1:**
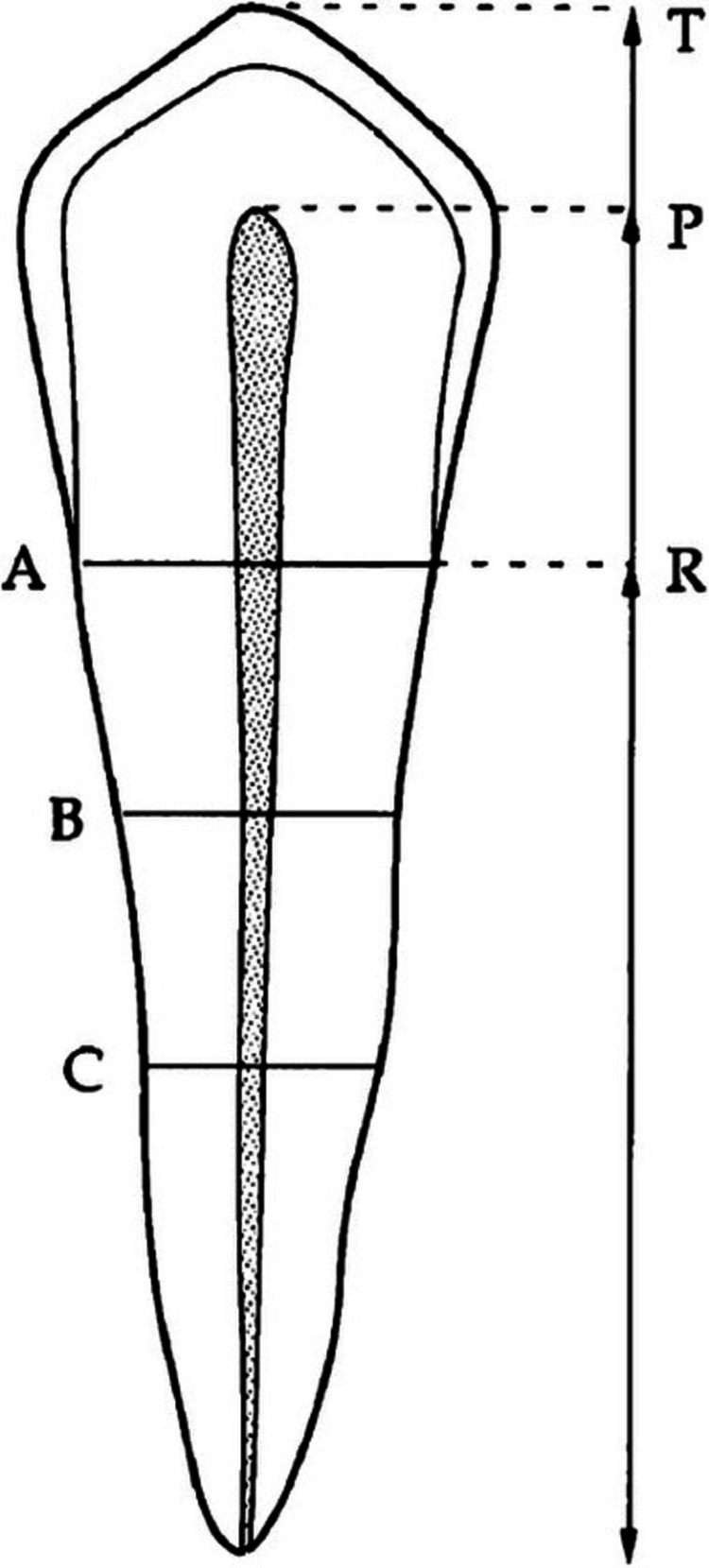
Measurements taken from each tooth

T represents the “Tooth and root length”; P represents the “Pulp and root length”; R represents the “Pulp and tooth length”; A represents the “Pulp and root width at a level”; B represents the “Pulp and root width at b level”; C represents the “Pulp and root width at c level” as shown in Figure 1. All of the above ratios were generated using Kvaal’s methodology. The data were analyzed through SPSS software (IBM Corp., Armonk, NY).

## Results

IBM SPSS Statistics Version 27 statistical analysis software (IBM Corp., Armonk, NY) was used for the statistical analysis. Statistical formulas, such as mean, standard deviation (SD), correlation, regression, and coefficient of determination, were utilized. The student's t-test was employed to determine the significance of the two means. P-values were classified as non-significant if they were greater than 0.05, significant if they were less than 0.05, and highly significant if they were greater than 0.01 (Table 1).

**Table 1:** Descriptive Statistics

Descriptive Statistics					
	N	Minimum	Maximum	Mean	Std. Deviation
Age	74	18.00	64.00	32.431	11.243
Valid N (listwise)	74				

This study includes a total of 74 subjects of age range between 18 and 64 years with a mean of 32 years. The standard deviation was 11.243. See Table 2.

**Table 2:** Age groups

		Frequency
Valid	<= 23.00	14
	24.00 - 31.00	20
	32.00 - 35.00	14
	36.00 - 43.00	15
	44.00+	11
	Total	74

The subjects were divided into five groups below 23 (18 subjects), between 24 and 31 (24 subjects), between 32 and 35 (12 subjects), between 36 and 43 (13 subjects), and above 44 (7 subjects). See Table 3.

**Table 3:** Pearson correlation T represents the “Tooth and root length"; P represents the “Pulp and root length”; R represents the “Pulp and tooth length”; A represents the “Pulp and root width at a level”; B represents the “Pulp and root width at b level”; C represents the “Pulp and root width at c level” #11 #21: Upper Central Incisors #12 #22: Upper Lateral Incisors #15 #25: Upper Second Premolar #42 #32: Lower Lateral Incisors #43 #33: Lower Canines #34 #43: Lower First Premolar

Tooth no	#11 #21	#12 #22	#15 #25	#32 #42	#33 #43	#34 #44
T	-.235**	-.324**	-.466**	-.546**	-.581**	-.421**
P	-.181**	-.590**	-.124**	-.624**	-.225**	-.526**
R	-.166*	.854**	-.540**	-.424**	-.424**	-.322**
A	-.122*	.476**	.405**	.377**	-.454**	-.644**
B	-.281**	.517**	.449**	.404**	.664**	-.533**
C	-.235**	.449**	.387**	.340**	.731**	-.868**
M	-.224**	.939**	.809**	.884**	.451**	-.692**
W	-.201**	.483**	.525**	.384**	.523**	.973**
L	-.193*	.932**	.963**	.955**	.438**	.440**
W_L	.095*	-.883**	-.866**	-.877**	-0.07248	-0.03782
**. Correlation is significant at the 0.01 level (2-tailed).						
*. Correlation is significant at the 0.05 level (2-tailed).						

The above correlation table values the Pearson correlation, which tells the strength and direction of a linear relationship between variables. It ranges from -1 to +1. When the value is close to -1, it shows a perfect negative correlation. When it's close to +1, it tells the perfect positive relation, but we say no correlation when it's zero or close to zero. When values -0.4 or +0.4, we say moderate correlation.

Following that, a regression test was used to create regression equations for age assessment. Equations of regression were created for all six teeth, three maxillary and mandibular teeth, and all six teeth taken together by keeping age as a dependent variable, M as the first predictor, and W-L as the second predictor. See Tables 4-6.

**Table 4:** Regression equation

	All six	Maxillary	Mandibular
M	-.465^**^	-.833^**^	-.766^**^
W	-.484^**^	-.765^**^	-.837^**^
L	-.725^**^	-.608^**^	-0.060
W~L	-.655^**^	-.764^**^	-.634^**^

**Table 5:** Regression equation

Teeth no	Regression Equation	R^2^
11/21	Age=93.36‑98.25(M)11+20.15(W~L)11	0.648
12/22	Age=33.3‑55.2(M)12−63.82(W~L)12	0.665
15/25	Age=73.45‑94.36(M)15−39.36(W~L)15	0.534
32/42	Age=76.46‑96.34(M)]42−34.21(W~L)42	0.447
33/43	Age=38.45‑83.76(M)43−73.23(W~L)43	0.536
34/44	Age=140.44‑140.45(M)54−21.46(W~L)44	0.634

**Table 6:** Regression equation

Teeth no	Regression Equation	R^2^
Maxillary teeth	Age=116.4‑123.57(M)MAX+6.45(W~L)MAX	0.653
Mandibular teeth	Age=45.34‑123.23(M)MAND−76.8(W~L)MAND	0.752
All six teeth	Age=78.24‑96.46(M)OVR−34.6(W~L)OVR	0.640

When three mandibular teeth were analyzed combined, the coefficient of determination R2 was the highest (0.752). There is no significant difference between the mean estimated age and chronological age when all study teeth were taken individually, three mandibular teeth were taken together, maxillary teeth were taken together, and all six teeth were taken together. See Tables 7-8.

**Table 7:** Difference between the mean estimated age and chronological age

Tooth	Mean of chronological age (mean±SD)	Mean of estimated age (mean±SD)	T	P
11/21	32.43 ±9.69	32.43 ±10.23	0.016	1.00
12/22	32.43 ±9.69	32.43 ±9.32	0.000	1.00
15/25	32.43 ±9.69	32.43 ±10.48	0.013	1.00
32/42	32.43 ±9.69	32.43 ±10.26	0.014	1.00
33/43	32.43 ±9.69	32.43 ±11.47	0.000	1.00
34/44	32.43 ±9.69	32.43 ±11.41	0.000	1.00
All six teeth	32.43 ±9.69	32.43 ±9.04	−0.536	0.83
Upper three teeth	32.43 ±9.69	32.43 ±9.16	−0.411	0.63
Lower three teeth	32.43 ±9.69	32.43 ±9.93	0.015	1.00

**Table 8:** Standard error of estimate

Standard error of estimate (SEE) in years for six teeth individually, all the six teeth together, three maxillary teeth, and three mandibular teeth	
Teeth	SEE (in years)
11/21	7.26
12/22	7.64
15/25	6.98
32/42	8.24
33/43	7.24
34/44	8.12
All six teeth	8.36
Maxillary teeth	7.73
Mandibular teeth	8.02

## Discussion

In forensic research, age assessment techniques based on morphological changes in teeth and bones [8-9] and DNA methylation [10] have been used. Age estimation with dental radiographs, in particular, is noninvasive and can be utilized for both the living and the dead. Most age estimations based on dental radiographs employ the decrease in pulp cavity due to aging, as described by Kvaal et al., utilizing periapical radiograph.

Though the Kvaal method was originally designed for periapical radiographs, its use for panoramic radiographs has been investigated.

Digital panoramic radiographs are commonly employed in dental practice because they allow for the acquisition of pictures of six teeth in one ray. There was no statistical difference between the assessed and chronological ages when the Kvaal approach was applied to Saudi members. The mean value of all the relative multitude of ratios (M), which could address the pulp's general size, was utilized to determine a moderate relationship to age. To infer regression equations, an analysis of regression was performed utilizing M and W L as the first and second indicators, separately. When three mandibular teeth were considered combined, the coefficient of determination (R2) was highest (R2 = 0.752).

Various factors could influence the discrepancies between the current study and other investigations. It is commonly known that different demographic and ethnic groups have different dental development, tooth size, and pulpal cavity form [11-13].

## Conclusions

To survey the relevance of the Kvaal strategy to Arabic populaces, the boundaries utilized in the initial Kvaal technique were evaluated utilizing computerized panoramic radiographs of Saudi people.

The current examination identified that mean ratios of each of the six teeth are better correlated with width ratio, and the best indicators for age assessment were "M" (mean worth, all things considered) and "W L" (contrast among "W" and "L"). By consolidating every one of the six teeth, one can acquire a more precise age estimation. The investigation's finding proposes that this technique is possibly dependent on the derivation of regression equations on digital panoramic radiographs. As per this investigation, a future examination should utilize an enormous example size with a good portrayal of tests from different age groups, nationalities, and genders.
